# Nanoparticle Delivery Systems in the Treatment of Diabetes Complications

**DOI:** 10.3390/molecules24234209

**Published:** 2019-11-20

**Authors:** Eliana B. Souto, Selma B. Souto, Joana R. Campos, Patricia Severino, Tatiana N. Pashirova, Lucia Y. Zakharova, Amélia M. Silva, Alessandra Durazzo, Massimo Lucarini, Angelo A. Izzo, Antonello Santini

**Affiliations:** 1 Department of Pharmaceutical Technology, Faculty of Pharmacy, University of Coimbra (FFUC), Pólo das Ciências da Saúde, 3000-548 Coimbra, Portugal; joanacampos92@gmail.com; 2 CEB—Centre of Biological Engineering, University of Minho, Campus de Gualtar, 4710-057 Braga, Portugal; 3 Department of Endocrinology, Hospital de São João, Alameda Prof. Hernâni Monteiro, 4200–319 Porto, Portugal; sbsouto.md@gmail.com; 4 Tiradentes Institute, University of Tiradentes (Unit) and Institute of Technology and Research (ITP), Av. Murilo Dantas, 300, Aracaju-SE 49010-390, Brazil; pattypharma@gmail.com; 5 Laboratory of Nanotechnology and Nanomedicine (LNMED), Institute of Technology and Research (ITP), Av. Murilo Dantas, 300, Aracaju 49010-390, Brazil; 6 Arbuzov Institute of Organic and Physical Chemistry, FRC Kazan Scientific Center, Russian Academy of Sciences, 8, ul. Arbuzov, Kazan 420088, Russia; tatyana_pashirova@mail.ru (T.N.P.); luciaz@mail.ru (L.Y.Z.); 7 Department of Organic Chemistry, Kazan State Technological University, ul. Karla Marksa 68, Kazan 420015, Russia; 8 Centre for Research and Technology of Agro-Environmental and Biological Sciences (CITAB-UTAD), Quinta de Prados, 5001-801 Vila Real, Portugal; amsilva@utad.pt; 9 Department of Biology and Environment, University of Trás-os Montes e Alto Douro (UTAD), Quinta de Prados, 5001-801 Vila Real, Portugal; 10 CREA-Research Centre for Food and Nutrition, Via Ardeatina, 546, 00178 Rome, Italy; alessandra.durazzo@crea.gov.it (A.D.); massimo.lucarini@crea.gov.it (M.L.); 11 Department of Pharmacy, University of Napoli Federico II, Via D. Montesano, 49, 80131 Napoli, Italy

**Keywords:** diabetes mellitus, insulin, nanoparticles, drug delivery systems, oral hypoglycemic agents

## Abstract

Diabetes mellitus, an incurable metabolic disease, is characterized by changes in the homeostasis of blood sugar levels, being the subcutaneous injection of insulin the first line treatment. This administration route is however associated with limited patient’s compliance, due to the risk of pain, discomfort and local infection. Nanoparticles have been proposed as insulin carriers to make possible the administration of the peptide via friendlier pathways without the need of injection, i.e., via oral or nasal routes. Nanoparticles stand for particles in the nanometer range that can be obtained from different materials (e.g., polysaccharides, synthetic polymers, lipid) and are commonly used with the aim to improve the physicochemical stability of the loaded drug and thereby its bioavailability. This review discusses the use of different types of nanoparticles (e.g., polymeric and lipid nanoparticles, liposomes, dendrimers, niosomes, micelles, nanoemulsions and also drug nanosuspensions) for improved delivery of different oral hypoglycemic agents in comparison to conventional therapies.

## 1. Introduction

Diabetes mellitus (DM) is an epidemic metabolic disease that compromises the quality of life of patients, with its numbers continuously increasing [1]. The World Health Organization (WHO) reported 1.5 million deaths each year in worldwide [2], being one the most frequent cause of mortality in developed countries [1,3,4,5,6]. The incidence of diabetes is expected to be about 366 million in 2030 due to many factors, especially attributed to the increased percentage of population older than 65 years and to the sedentary lifestyle [7,8,9]. This disease is dependent on several factors (e.g., stress, low physical activity, unhealthy food habits, obesity, genetics, age, and inflammation), but some measures (e.g., weight reduction and diet intervention, monitoring of blood glucose and cholesterol levels and of blood pressure, exercise) can be taken to control the disease and its complications [3,4,10]. The treatment has to be based on glycemic control, balancing benefits versus drug’ secondary effects, considering that current glucose-lowering medications do not cure DM [11]. To control diabetes, simpler and painless routes for insulin administration are still in demand. Conventional drug delivery systems still have several limitations, e.g., improper and/or ineffective dosage, low potency, limited specificity for the target, which may result in adverse side effects in other organs/tissues [12,13,14]. Similar limitations are also encountered when using natural products of nutraceutical interest in the treatment of DM [15,16,17,18,19,20,21,22]. The loading of insulin, and other sugar-lowering drugs and nutraceuticals [23,24,25,26,27], into nanoparticles has been proposed as a more convenient, non-invasive and safer approach through alternative administration routes.

A number of nanoparticle-based delivery systems have been proposed to overcome the enzymatic degradation in the stomach and thus to enhance permeation through the gastrointestinal tract (GIT), with the aim to improve oral insulin absorption [11]. DM is characterized by an inadequate insulin production (pancreatic islet cells destruction) and also by a lack of sensitivity of host cells to endogenous insulin, being classified into Type 1 DM (T1DM) and Type 2 DM (T2DM) [1,3,4]. T2DM is responsible for more than 85% of total diabetics and is characterized by an impairment of pancreatic beta cells in the pancreas of patients, which compromise the capacity of the organism in using insulin. T1DM, on the other hand, is an autoimmune disorder, in which pancreatic β-cells are attacked, reducing or impairing insulin production [11]. In both types of diabetes, it is necessary to reduce the rate of disease progression and ameliorate the symptoms of hyperglycemia, and its associated complications [28,29]. In order to manage the blood sugar level (BSL) in both types of DM patients insulin is used [3,4]. In T1DM patients, insulin is used as a conventional therapy, but it is also used in T2DM patients at a later stage, i.e., when glycemic control does not respond to diet, regular exercise or weight loss and nor to oral antidiabetic agents [11]. Currently, to replace insulin in T1DM subcutaneous insulin injection or surgical implantation of β-Langerhans cells are the only available approaches [28,29]. In the case of T2DM, to dominate BSL and attenuate long-term complications, oral antidiabetic drugs and insulin replacement therapy are commonly used [30,31]. Insulin replacement has however several disadvantages, including the discomfort of subcutaneous injection and the limited patient’s knowledge of glucose levels [29,32].

Due to its function in diabetes in the control the blood glucose levels, insulin is a life-saving drug. This 5800 Da polypeptide is a hormone with a chain of 21 amino acids and other of 30 amino acids that are joined by two disulfide bonds, with an active role in the glucose uptake and storage in the liver and muscles [33,34]. Its structure (monomers, dimers, tetramers and hexamers) varies in solution depending on the composition of the solvent media and easily shapes fibril at acidic pH and elevated temperatures, in the presence of organic chemicals and under vibration [35].

When the blood glucose levels are elevated, the β-cells of islets of Langerhans produce insulin in the pancreas, which is then released in the blood stream by exocytosis in order to promote the utilization of peripheral glucose to generate energy [11,34]. After it is secreted into the portal vein, it is transferred to the liver where is subject to the hepatic first-pass effect (almost half of the polypeptide hormone experiences hepatic degradation), creating an insulin concentration gradient between hepatic portal and systemic circulation, i.e., in the liver insulin concentration is higher than in the peripheral systemic circulation [36,37]. The generation of reactive oxygen species (ROS) also plays a role in DM, i.e., the increased oxidative stress and thus the decrease of the antioxidant potential contributes to the secondary diabetic complications. Insulin controls the metabolism of carbohydrates and lipids (i.e., synthesis of glycogen in the liver and lipids degradation in adipose tissue) and also represses hepatic gluconeogenesis, protein breakdown and autophagy, as well as it boosts uptake of potassium in the peripheral tissues and sodium reabsorption in the kidneys [11]. There are many anti-diabetic drugs (e.g., metformin, gliclazide, GLP-1 analogues and DPP4 inhibitors), which when taken with food stimulate insulin’s receptors and stabilize glucose levels (plasma glucose 70–140 mg/dL or glycated hemoglobin (HbA1c) about 7%) [38].

In T1DM patients, the main treatment for maintaining blood glucose levels is the administration of exogenous insulin by subcutaneous injection, which is a mixture of rapid-acting human insulin (aspart, glulisine and lispro) or immediate-acting (neutral protamine Hagedorn insulin and Lente insulin) to reset blood glucose after meals and long-acting insulin (glargine detemir) before bed to maintain basal level of insulin in the circulation during the day [39].

Oral insulin administration is known to result in low bioavailability and insufficient therapeutic effect, due to the physiological instability of the polypeptide attributed to its chemical and enzymatic degradation in the GIT, and to the fast systemic clearance [40]. Conventional insulin has therefore to be administered daily through several subcutaneous injections, which cause patient’s discomfort, pain, stress and trauma, resulting in limited compliance. Insulin injections are also associated with the risk of local infections, hypoglycemia, skin necrosis and nerve damage [11,41]. Endogenous and exogenous insulin have different pharmacokinetics, as the latter acts in peripheral tissue without undergoing liver metabolism. The activity of insulin (in the skeletal muscle, liver, adipose tissue and kidneys) is compromised by the insulin resistance pathways that aggravate its secretion and resistance, either by deposition of fatty acids in skeletal muscles or by peripheral hyperinsulinemia [11]. To overcome insulin resistance, several non-invasive routes have been proposed, among which oral insulin delivery stands out as due to patient’s compliance and higher comfort. Besides, it allows rapid hepatic insulinization (avoiding gluconeogenesis in the liver) and also avoids peripheral hyperinsulinemia or another secondary effects, making it a convenient route, for example, for chronic disease [28].

A diversity of approaches (e.g., use of permeation enhancers, chemical modifications, enteric coatings, enzymatic inhibitors and nanoparticles) has been exploited to optimize the oral delivery of insulin. Nanoparticles (i.e., particles with a size range between 10^−9^ and 10^−7^ m) composed of different materials have been successfully used for the loading of antidiabetic drugs (curcumin and berberine) with superior outcomes in vivo in the control over the blood glucose levels [42].

It has also been demonstrated the possibility to modulate the targeted delivery by the coating of nanoparticles with site-specific ligands [11]. Clinical trials with nanomedicines, in Europe, have increased; studies on follow up, use, and compliance are however still needed as reported by recent studies in the area [23,24,25,26] and in assessment strategies [26].

This review discusses the advantages of using nanocarriers (e.g., enhanced the solubility of poorly water-soluble sugar-lowering drugs, improved stability of insulin, increased absorption/permeation of loaded anti-diabetic drugs resulting in higher bioavailability) for the delivery of anti-diabetic drugs in the management of DM for different administration routes.

## 2. Administration Routes

Conventional drug delivery systems are commonly associated to low efficacy (due to improper or ineffective dosage), low potency and altered effects (drug metabolism and lack of target specificity) [27]. As current insulin formulations do not mimic the physiology of the human insulin, which is endogenously secreted, the development of more physiologically friendly formulations to be administered by less invasive routes (such as oral, transdermal, inhaled and nasal, buccal, ocular and rectal) is still in demand. Various types of nanocarriers have been tested to deliver anti-diabetic drugs, but although stability and therapeutic efficacy of these new formulations have been taken into account in their development, the results on the bioavailability are still behind expectations [37,43,44]. Due to the lack of stability through other administration routes, protein drugs are delivered by injection to ensure their bioavailability and efficacy [11,37]. However, multiple daily insulin injection is associated with local infection, hypertrophy, fat deposits at injection sites, and trypanophobia [41]. Although exogenous insulin can reduce morbidity and mortality, for all the reasons explain above, about 60% of diabetic patients cannot maintain long-term normoglycemia. Thus, low patient compliance does not allow the glycemic control and contribute to diabetic ketoacidosis, taunting micro- and macrovascular complications [37].

Eye’s anatomy and physiology barriers (blood-aqueous and blood-retinal barrier) limit the delivery of drugs by ocular route. Diabetes has some complications, among which neuropathy and nephropathy stand out, as well as diabetic retinopathy (DR) [45]. DR, the most common disease of the posterior segment, is usually treated by laser surgery, or through local eye injections of anti-vascular endothelial growth factor (anti-VEGF) as only about 2% of the drug reaches the back of the eye if administered through other systemic routes (e.g., oral, intravenous or subcutaneous) [5,6,46,47]. For the treatment of ocular diseases, systemic administration is not recommended, being the periocular administration (peribulbar, posterior juxtrascleral retrobulbar, subtendon and subconjunctival route) the most promising and efficient route to achieve and maintain the therapeutic concentration without causing severe side effects in the intraocular tissue [48]. Periocular administration of drugs is however an invasive procedure and is associated with fast systemic clearance. In addition, the barriers encountered by the drug molecule to reach the intraocular tissues compromises its bioavailability thereby requiring frequent administration, which further increases the cost of the treatment. The frequency of intravitreal injection has numerous complications, as retinal toxicity, damages in the retina and lens, endophthalmitis, high intraocular pressure (IOP) and inflammation, retinal detachment and intraocular lens dislocation, cataract, hemorrhage, retinal vascular occlusion, cystoid macular edema, and hypotony [6,49]. To overcome the ocular barriers and increase patient’s compliance, non-invasive and sustained release alternatives that prolong the contact time of the drug with the tissues, enhancing its bioavailability have been proposed [50]. Preclinical and clinical studies have been run to evaluate the potential use of nanoparticles for the site-specific targeting to the posterior segment of the eye [51,52,53].

Nanoparticles allow the controlled release of the drug into intraocular tissues without invasive approaches. As the retention time is enhanced, the amount of drug being absorbed also increases as well as its bioavailability. Nanoparticles can be used for site-specific targeting thereby minimizing the risk of adverse side effects. Indeed, advanced nanoparticles are being used for ocular drug delivery, as they can overcome the eye barriers mainly due to their unique physicochemical properties and when it compared to the classical pharmacotherapy can improving the therapeutic efficacy [54,55,56,57,58]. If applied topically onto the eye sclera, nanoparticles can be retained onto the surface of the eye offering resistance against lacrimal fluid and blinking, and acting as drug depots. This is particularly significant if cationic nanoparticles are used [47,59,60].

The first results on oral insulin were obtained in 1922, up to now oral insulin formulations have not yet been successful [37,61]. The orally administered insulin would be the approach that best mimics the normal physiological pathway, leading to better homeostasis, besides enhancing the patient’s compliance. Insulin is absorbed in the intestinal lumen, reaching the liver (final target) via portal circulation, therefore creating a high porto-systemic gradient. Due to the lower levels of systemic insulin, the administration of the peptide by oral route would result in lower risk of hypoglycemic events, as well as weight gain problems. The liver is extremely responsive to insulin, in which the glucose production is regulated by the concentration of the peptide; glucose production stops when the portal insulin concentration reaches 50 μU/mL in healthy subjects and 100 μU/mL in diabetic patients. Oral insulin has a protective role in β-cells of the pancreas due to autoimmune destruction and can overcome the slow release kinetics encountered in the first phase of subcutaneous administration [37]. While the oral route has the highest patient’s compliance, the oral administration of anti-diabetic peptides is hindered by the chemical and enzymatic degradation in the GIT as well as by epithelial cell monolayer in the its membrane, resulting in low bioavailability [62,63].

**Figure 1:**
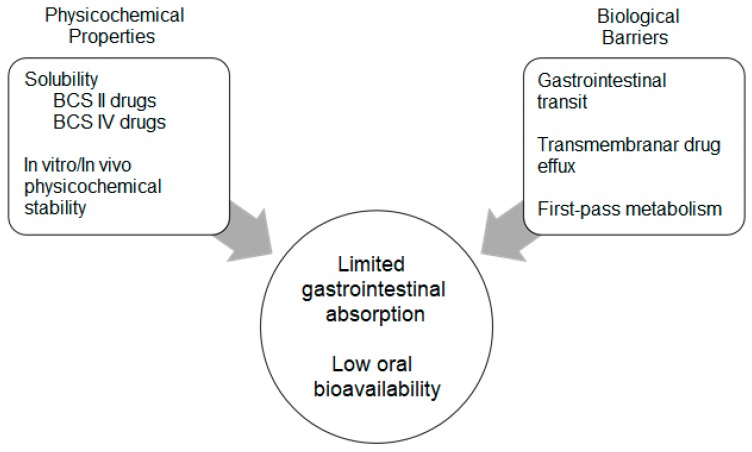
Challenges encountered in oral drug delivery.

There are several challenges encountered in oral drug delivery (Figure 1). After oral administration, drugs have to overcome stomach, intestinal lumen, mucus membrane of the intestinal epithelium as well as the epithelium itself. The stomach is characterized by four layers (mucosa, submucosa, muscularis externa and the serosa) and it is coated by a mucous membrane that allows the secretion of gastric fluid. The apical surfaces of enterocytes (abundant absorptive cells in the intestine) are covered by rigid, finger-shaped and negatively charged microvilli that increase the surface region available, increasing the drug absorption. The gap between microvilli is less than 25 nm, inhibiting the passage of large particles. The characteristics of the GIT, mainly tight conjunction, turnover of epithelial cells and also low leakage, form a barrier against the paracellular transport of insulin. Its physiology, mainly poor mucosa permeability and also drug’s degradation before absorption, can cause poor absorption and low drug bioavailability [62]. Effective oral delivery of insulin features physiological barriers, namely physical, biochemical and enzymatic barriers. The intestinal epithelium layer is composed of enterocytes, goblet cells, Paneth and microfold (M) cells, which offer defense tools against chemicals and pathogens. M cells (rich in Peyer’s patches), either by endocytosis of the antigens or by successive immune response, can generate mucosal immunity. Due to its molecular weight and hydrophilic properties, insulin cannot cross the GIT, besides it is difficult for drugs to escape from the enterocytes via transcellular route [11,37]. Thus, the delivery of hydrophilic and sensitive peptide drugs is still a challenge, being their loading into new drug delivery systems (e.g., nanoparticles) an alternative to overcome these limitations, mainly the acidic pH environment and the digestive enzyme degradation [64,65,66,67,68].

Nanoparticles may be used to increase the paracellular absorption of drugs. Nanoparticles with hydrophobic surface are advantageous for epithelial endocytosis. On the other hand, cationic nanoparticles interact with the negatively charged mucus layer limiting their absorption [69]. At the same time, the secretory goblet and also Paneth cells protect and repair the intestine, through the production of mucins and antimicrobial peptides. Nanoparticles developed for oral insulin delivery have to overcome mucus and epithelial barriers. Nanoparticles of neutral and hydrophilic surfaces are preferential to overcome mucus, but their interaction with epithelial cells may be compromised [11]. The low oral bioavailability found to be due to the poor permeation through the intestinal epithelium is also attributed to pre-systemic chemical degradation in the stomach. Stomach’s lumen is extremely acidic (pH 1–3.7) and in the intestine the pH varies between neutral/alkaline range (pH 6.5–8), which can tease pH-induced oxidation and deamination of protein drugs [11,37]. An oral dosage form has to consider the transit time (e.g., for a tablet is ca. 2.5 h in the stomach and 3–4 h in the intestine), it has to resist to the low pH in the stomach, and insulin has to keep the tertiary structure and its biological activity along the GIT [11]. While nanoparticles loading insulin survive the pH variations of the GIT, their paracellular and transcellular absorption is still influenced by digestive enzymes (endopeptidase trypsin, alpha-chymotrypsin, elastase and carboxypeptidase A and B). The proteolysis of insulin oral dosage forms occurs in the stomach and follows intracellular peptidase degradation in the enterocytes. In a study made in the small intestine of rat, an amount of insulin was degraded in the mucus layer (duodenum > jejunum > ileum). The enzymes responsible for catalysis and breakdown of the absorbed insulin in the systemic circulation are the liver conjugation enzyme and cytochrome P450 (CYP) enzymes [11]. Trypsin, α-chymotrypsin and carboxypeptidases in the mucous layer and intestinal lumen, as well as a specific insulin-degrading enzyme (IDE) on the brush-border membrane are responsible for the degradation of insulin [37].

Other insulin administration routes (pulmonary and nasal delivery) have also been previously studied and are already marketed (Exubera^®^ and Afrezza^®^). These administration routes also have some drawbacks such as lack of patients compliance, variation of absorption in alveoli surface and the need of appropriate handling inhalation technique [41].

## 3. Nanoparticles for Oral Delivery of Anti-Diabetic Drugs

**Figure 2:**
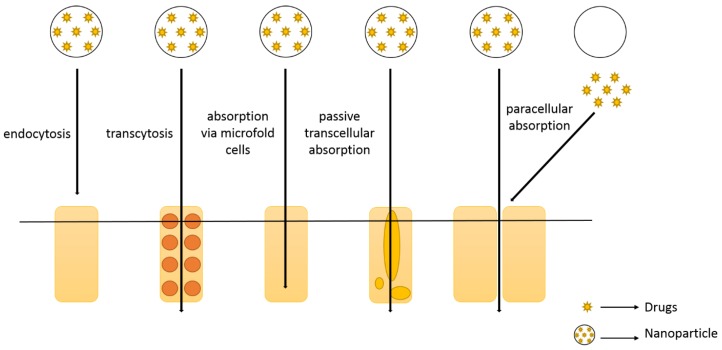
Scheme of various pathways for the oral absorption of nanoparticles through the gastrointestinal tract.

The loading of drugs into nanoparticles can improve the stability of the former, protecting them against chemical and/or enzymatic degradation in the GIT. Nanoparticles also increase the contact with the GI epithelium, increasing the residence time, drug absorption and bioavailability. Drugs can either be entrapped within the nanoparticles matrix or attached onto their surface, and they have to be released near of the absorption site [62]. An ideal nanoparticle should enter through the GI membrane, and for that the nature of the selected polymer, the mean particle size and polydispersity, the surface electrical charge, hydrophilicity and morphology of the particles are instrumental for the nanoparticles uptake in the GIT [70]. Nanoparticles should also demonstrate prolonged circulation time, increased mean residence time (MRT) and reduced clearance [62]. Lymphatic uptake of drugs is an alternative to overcome the first pass metabolism. Although the lymphatic system has a channel network along the body, it is a one-way passage, being considered an efficient route for oral drug delivery across the intestinal membrane. Nanoparticles are transferred into the lymph and reach the lymphatic system via M cells [71,72]. Figure 2 summarizes different routes for the oral delivery of nanoparticles.

Over the last decade, nanoparticles have been studied for oral administration of insulin [11,64,65,66,67,68,73,74]. Nanoparticles are the delivery systems that have the largest volume/size ratio in comparison to other dosage forms, being that have been explored various natural polymeric, synthetic polymeric as well as inorganic nanoparticles [11].

### 3.1. Natural Polymeric Nanoparticles

#### 3.1.1. Chitosan-Based Nanoparticles

Chitosan, a natural polymeric polysaccharide, is composed of deacetylated glucosamine and *N*-acetyl-d-glucosamine and show ideal properties (biocompatibility, biodegradability, mucoadhesiveness, permeation enhancing effect and non-toxicity) for in vivo administration. The deacetylation’s degree from chitin and molecular weight, as well as the pH value are responsible for the physiochemical properties of chitosan that has a positive surface charge, forming hydrogen and electrostatic ionic bonds to the sialic-acid group in mucin [64,66,67,73,74]. Chitosan nanoparticles have mucoadhesive properties in the GIT, which has also been studied at the molecular level [75]. Chitosan has the ability to re-distribute of claudin-4 (CLDN4) from the cell membrane to the cytosol, degrade lysosome and weaken the tight junctions, and enhances paracellular permeability. For chitosan treatment, junctional adhesion molecule (JAM-1) translocation as well as zona occludens-1 (ZO-1) disappearance were also reported [76]. Insulin could be protected against enzymatic degradation using formulations combining chitosan nanoparticles and oleic acid. These nanoparticles were administered to rats with free access to food; however, the reported low oral bioavailability was attributed to the enhanced residence time of the particles in the presence of food, resulting in the degradation of insulin [77,78].

There are several pH-responsive nanoparticle delivery systems (cationic chitosan and anionic γ-PGA) designed for oral delivery of insulin [11]. Simple ionic gelation method has been used without the need of harmful organic solvents or sonication. It was proven in a study that manipulating the polyelectrolyte complexation technique, insulin loading was higher in comparison to the ionic gelation method [79]. Negatively charged insulin can interact with positively charged polymers through polyelectrolyte complexation technique. Using the ionic gelation method, insulin was entrapped in the nanoparticle matrix, diffusing the drug to the external medium. Excipients (magnesium sulphate (MgSO4) and sodium tripolyphosphate (TPP)) allow a higher stability of nanoparticles at 2–7.2 pH range, improved drug loading as well as paracellular permeation effect [80,81,82]. As TPP is negatively charged, it forms a matrix through the interaction with positively charged polymers [79,83,84,85]. The pKa values of chitosan (amine group) are 6.5 and γ-PGA (carboxylic group) are 2.9, thus the polyelectrolyte complex does not degrade at 2–7.2 pH but disintegrates above pH 6.5 [86]. It was proven that the insulin-loaded nanoparticles reduce in 50% the BSL while the hypoglycemic effect was kept after oral administration in a diabetic rat model [81]. When insulin is released from the nanoparticle matrix, it is exposed to the intestinal enzymes. In order to attenuate the insulin degradation by protease, a complexing agent—diethylene triamine penta-acetic acid (DTPA)—is used as an excipient capable of depriving Ca^2+^ and Zn^2+^ from the intestinal enzymes [81,87]. The use of a complexing agent in chitosan nanoparticles improves insulin absorption by chelating the extracellular Ca^2+^ and by modulation of the tight junction [88,89]. The proposed delivery system improved the oral bioavailability of insulin by 20% attributed to the enzyme inhibitory activity and enhanced permeation effect. However, this inhibitory effect, as it is prone to extensive GI fluid dilution, is dependent on the concentration of the complexing agent [81]. To take advantage of Ca^2+^-binding specificity, the ethylene glycol tetra-acetic acid (EGTA) was used in diabetic rats exhibiting a protective effect against protease and higher hypoglycemic effect in comparison to DTPA [89]. No clinical manifestation over the 14 days of treatment with nanoparticles were reported in an in vivo study, together with no bacterial invasion even though the epithelium has been temporarily discontinued [90]. Moreover, lyophilizing the chitosan-γ-PGA nanoparticles prevented the release of insulin in the stomach. The nanoparticles were packed in a gelatin capsule, covered with Eudragit^®^ S100 or L100-55, as well as dissolving at pH above 7 (jejunum and ileum pH) and pH 6.6 (pH of the duodenum), respectively [91]. In this formulation, in the freeze-drying process trehalose was used as cryoprotectant, to stabilize the nanoparticles in a glassy matrix, protecting them against freezing stress that occurs in the preparation process. Other chitosan-based pH-sensitive nanoparticles have also been studied, including carboxylated and trimethylated (TMC), ethyl (DMEC), carboxymethyl (CMCS) and also thiolated chitosan. In an acidic environment below pH 6.5, dissolution of the matrix of chitosan nanoparticles occurs through protonation of amine group [92]. It is known that, after dissolution studies, 60% of insulin is released in hydrochloric acid (pH 2) due to the penetration of pepsin into the surface of nanoparticles [93]. In the duodenum (pH 6.6), chitosan properties become compromised; as it is water insoluble, it deprotonates, precipitates, loses some of its mucoadhesive properties while intracellular tight junctions also loose capability in the alkaline environment [94]. Chitosan derivatives (carboxylated chitosan, TMC, DMEC, CMCS and thiolated chitosan) are then used in nanoparticles formulation to enhance the water-solubility, mucoadhesive ability and penetration properties in alkaline pH [11]. Based on pH and also due to the enzymatic instability in the GIT, a colon targeted drug delivery system has been proposed [95]. Using chitosan free polymers co-formulated with chitosan derivative-based nanoparticles it is possible to improved oral insulin bioavailability and decreased BSL effect. In comparison to conventional chitosan, TMC protonates more easily its amine group in neutral or alkaline environment, which improves the water solubility and permeation of peptides [93]. TMC-γ-PGA nanoparticles proved to be more stable; 30% of the encapsulated insulin was released at pH 7.0 and the swelled matrix showed a sustained release of insulin at pH 7.4 [96]. TMC-γ-PGA-nanoparticles are more convenient for transmucosal insulin delivery within the entire intestine, while chitosan-γ-PGA nanoparticles dissociated in the duodenum and showed paracellular absorption. However, these mucoadhesive properties can influence endocytosis absorption, since most of nanoparticles were retained in the mucus layer being removed together with detached mucus [97]. It is known that insulin is negatively charged and its electrostatic interaction with positively charged TMC results in rapid release of insulin. As previously discussed, orally administered nanoparticles require specific properties to permeate the mucus and the epithelial layer. To overcome these barriers, some approaches have been proposed. Firstly, nanoparticles were coated with poly(ethylene glycol) (PEG) to create a hydrophilic and neutral surface and thus increased the penetration in the mucus layer. The developed PEG-modified chitosan nanoparticles improved the stability of insulin, reduced hemolysis, thrombosis and embolization, but the cellular uptake in the GIT was hindered by the hydrophilic surface of the particles. Secondly, CSKSSDYQC (CSK) targeting peptide has elevated affinity for the goblet cells producing the mucus. By combining TMC with CSK the uptake of nanoparticles by villi was promoted, permeation of insulin was facilitated, and active absorption of insulin was enhanced via clathrin and caveolae endocytosis [11]. Assemblies of graft copolymer micelles (dodecylamine-graft-g-polyglutamic acid (PGA-g-DA)) loading insulin and TMC-CSK nanoparticles, improved the stability of the peptide, protecting it from enzymatic degradation thereby improving its intracellular/transcellular uptake [98]. Nanoparticles composed of TMCpHPMA copolymers were orally administered to diabetic rats placed in a diffusion chamber [99]. Nanoparticles showed mucus permeation while the insulin release seen at pH 2 suggests that this carrier can be surface tailored with an enteric coating. Chitosan’s thiolated groups can stimulate the interaction with cysteine residues of mucus glycoprotein, showing to be more mucoadhesive in comparison to control chitosan nanoparticle-loaded tablets. The tablet formulation decreased the BSL, however the pharmacological efficacy was lower than that observed after subcutaneous injection [100]. Other formulations combining TMC and thiomer or cysteine have been proposed to increase the permeability of insulin [11,101]. Layer by layer (LBL) coated nanoparticles were reported to be a suitable alternative to improve insulin stability at gastric pH and its oral bioavailability [102]. To prepare nanoparticles, this type of coating does not require organic solvent or high temperature, which could compromise the bioactivity of the peptide, but it is a time-consuming approach because of the multiple adsorption layers and centrifugation processes. A study reported the advantages of LBL coated nanoparticles for oral delivery of insulin where vitamin B12 (known to increase GI absorption and plasma residency time) was conjugated with chitosan and also alginate to form a nano-complex (VitB12-Chi-CPNPs) [103,104]. VitB12-Chi-CPNPs binds to the intrinsic factor (IF) receptor (cubulin), a glycoprotein secreted by the parietal cells of the gastric mucosa, forming a complex, undergoing receptor mediated endocytosis by intestinal enterocytes. In neutral or alkaline environment, this complex enhanced the water solubility [105]. After incubation in caco-2 cell monolayers, the formulation showed some insulin transport by natural uptake of Vitamin B12 (transcellular and paracellular absorption). Despite the delayed BSL reduction, this complex showed a sustained hypoglycemic effect in vivo.

The use of PEG chains lower than 5 kDa may contribute to increase mucus diffusion of particles [106,107].

Glucose sensors (glucose oxidase, boronic acid, 4-formylphenylboronic acid, poly(3-methacrylamido phenylboronic acid) (PMAPBA), boronic acids-(2-formyl-3-thienylboronic acid (FTBA) and 4-formylphenylboronic acid (FPBA)) have also been conjugated with chitosan nanoparticles for glucose-dependent insulin release [11].

#### 3.1.2. Alginate-Based Nanoparticles

Alginate, an anionic polysaccharide, is composed of alternating blocks of (1–4)-linked β-d-mannuronic acid and α-l-guluronic acid residues [108]. It is a hydrophilic, biodegradable and biocompatible, a mucoadhesive and pH sensitive polymer. This polymer allows insulin retention in the nanoparticles, due to its guluronic acids residues, which, by exchanging sodium ions, can bind with divalent ions to form a gel matrix [109]. The α-helix and β-sheet of insulin structure were kept when the peptide was loaded into alginate-chitosan nanoparticles produced by ionotropic pre-gelation, which can further be reinforced using glutaraldehyde and divalent ions (Ca^2+^) [110]. To further increase its stability, insulin can be conjugated with cationic β-cyclodextrin polymer (CPβCDs) by electrostatic interaction prior to the encapsulation in alginate-chitosan nanoparticles [111]. These particles however were not appropriate for modified release of the protein as more than 40% was released in simulated gastric fluid (SGF) [111].

#### 3.1.3. Dextran-Based Nanoparticles

Dextran is a hydrophilic, biodegradable, biocompatible, negatively charged polysaccharide, capable of linking with positively charged chitosan and proteins. It has been proposed to load insulin in alginate-dextran nanoparticles prepared by the nanoemulsion dispersion method and in situ gelation [112]. At pH 1.2, alginate limits insulin release by creating a compact matrix whereas at pH 7.4 the matrix becomes leaky releasing the peptide. The oral administration of dextran sulfate-chitosan nanoparticles containing insulin reduced the BSL. In the same way, multilayered nanoparticles based on the LBL technique (alginate, dextran sulfate, chitosan and albumin) were also reported to be resistant to acidic pH, reducing the basal blood sugar level and increasing insulin bioavailability [113]. Vitamin B12-coated dextran nanoparticles loading insulin reduced BSL and hypoglycemic effect in diabetic rats [114,115]. To increase the encapsulation efficiency of insulin of these particles, ternary inter-polyelectrolyte complex of insulin, dextran sulfate and poly(methylaminophosphazene) (PMAP) has been proposed [116]. This complex enhanced the protection of loaded insulin against gastric proteolytic enzyme, which could then be released under simulated intestinal environment (SIF) [11].

### 3.2. Synthetic Polymeric Nanoparticles

#### 3.2.1. PLGA Based Nanoparticles

PLGA, a synthetic polymer, is known for its biodegradability and biocompatibility, being one of the most popular polymers for the production of nanoparticles for drug delivery [51,54,117,118,119,120,121,122]. PLGA nanoparticles are usually negatively charged, which causes poor penetration through the mucus layer, which is also negatively charged. To improve the loading capacity of insulin in PLGA nanoparticles, Cui et al. proposed the formation of an insulin-phospholipid complex followed by the production of PLGA nanoparticles by reverse micelle-solvent evaporation [123]. The obtained nanoparticles were exposed to acidic stomach and digestive enzymes. No burst release of insulin was reported in vivo. To stabilize insulin at low pH, modification of the nanoparticle surface, use of enzyme inhibitors or enteric coating have been proposed. To enhance absorption in the epithelial membrane, hydrophobic ion pairing method has been used to improve hydrophobicity of insulin [124]. A complex between sodium deoxycholate and insulin was loaded in PLGA nanoparticles by emulsion solvent diffusion method, improving the encapsulation efficiency of the peptide and given to diabetic rats, with reduced BSL [125,126].

To allow sustained release of insulin in the GIT, a star-branched β-cyclodextrin-PLGA copolymer was used to improve the pH sensitivity [127]. The nanoparticles were prepared by a modified double-emulsion method with high insulin encapsulation efficiency. Low insulin was released at neutral pH. Another study evaluated the potential of pH-sensitive cellulose derivative (HPMCP-55) as a component of PLGA nanoparticles (PLGA-Hp55) to improve the oral delivery of insulin [128]. In simulated gastric fluid, PLGA-Hp55 nanoparticles delivered less insulin in comparison to PLGA nanoparticles. The use of HP55 in the nanoparticles can depress initial BSL. A PLGA/Eudragit^®^ RS nanoparticle delivery system coated with HPMCP-55 enhanced enzymatic protection of insulin and its bioavailability [129]. It is thought that neutral and hydrophilic nanoparticles are ideal to overcome the mucus barrier. However, in a study in HT29-MTX cells, the effect of TMC coating on PLGA nanoparticles was evaluated, showing that the positively surface charge facilitated the penetration of nanoparticles in the mucus [130]. In comparison to uncoated PLGA nanoparticles, TMC-PLGA nanoparticles showed higher diffusion. It was proven that the association of chitosan derivatives with the mucus layer (electrostatic interactions, hydrophobic forces, van der Waals forces and hydrogen bonds) was fundamental in insulin GI absorption. In diabetic rats after oral administration, TMC-PLGA nanoparticles were absorbed in the GIT by transcellular (clathrin-mediated endocytosis) and also paracellular pathway (tight junction opening), with glucose-lowering effect [11].

To improve the GIT mucoadhesiveness of PLGA nanoparticles, cationic chitosan has been proposed to be used as coating to modify the surface charge of the particles [131]. Another nanoparticle formulation, conjugating folate and PEGylated PLGA, was used to enhance GI uptake of insulin via M cells and folate receptors [132]. In dissolution study of this formulation, initial burst release was observed in alkaline environment and only about half of accumulated insulin released. To improve the insulin release profile in SIF, additional optimization should be done. To promote intestinal absorption of insulin cell-penetrating peptides (CPP) can also be used. CPPs (d-arginine octamer, L-penetratin, L-pVEC and L-RRL helix) are known by improving insulin stability and absorption, being capable to penetrate in the cell membranes of the GIT [133]. In comparison to non-modified PLGA nanoparticles, the cellular internalization, bioavailability and hypoglycemic effect of poly(arginine)8 enantiomers (L-R8 and D-R8) modified nanoparticles were described. However, additional studies will have to be done to understand the mechanisms of GIT internalization for these nanoparticles. Concanavalin A (Con A), a lectin that recognizes and binds carbohydrate moieties of cells, when lectins bind to intestinal mucosa, biological signals are transmitted to enterocytes, triggering endocytosis for GI uptake of orally administered peptide. ConA-modified PLGA can overcome the first-pass metabolism in the liver by improving oral bioavailability through M-cell transcellular absorption [134,135]. In another study, Con A-modified PEGylated PLGA (PPC) also improved the stability of nanoparticles, with enhanced transport through the rat intestinal wall, higher oral absorption by increasing lymphatic uptake in the GIT, as well as slow clearance in the blood circulation [136]. When PPC nanoparticles were administered in diabetic rats, a delayed BSL reduction was shown. In a study that conjugated l-valine with PLGA nanoparticles, there was also an increase insulin absorption by lymphatic uptake and transport through small intestine against concentration gradient [137]. In comparison to unmodified PLGA-nanoparticles, l-valine conjugated PLGA nanoparticles had more insulin transported through intestinal mucosa, and enhanced hypoglycemic effect. Currently, transferrin (Tf) is being used to coat the surface of PLGA nanoparticles to facilitate insulin absorption in the GIT [138]. Poly(ester amide)s (PEAs) were used as the nanoparticle polymer while arginine was used as the cationic component. In diabetic rats, these Tf-coated PLGA nanoparticles loading insulin caused BSL reduction [11].

#### 3.2.2. Poly(Lactic Acid) (PLA)-Based Nanoparticles

PLA, a biodegradable and biocompatible polymer, is usually used for oral drug delivery. Polymeric PLA-b-pluronic-b-PLA (PLAF127-PLA) form a vesicle that was employed for oral insulin delivery [139]. Although these nanoparticles induced hypoglycemic effects, initial burst release of insulin was observed, causing local toxicity to the GIT. PLA-PEG nanoparticles were modified, attaching IgG Fc fragments to target the neonatal Fc receptor (FcRn) [140]. Adult FcRn transport IgG antibodies through small intestine and colon, being expressed at similar level to fetal expression [141]. These nanoparticles move through rat intestinal epithelium to reach blood circulation. In comparison to other oral insulin delivery systems, Fc modified PLA-PEG nanoparticles required few insulin doses to show a hypoglycemic effect. They have also minimal interruption of GIT associated with the paracellular or transcellular pathway, and high expression of FcRn along the intestine. However, to explore the impact of IgG Fc fragment on immunological response and tissues expressing FcRn further studies are still needed. Nanoparticles coated with dilauroylphosphatidylcholine zwitterions exhibited electrically neutral and hydrophilic shell, and allow protection of negatively charged hydrophobic PLA core [142]. A uniform GI distribution of the nanoparticles and improved intestinal peptide transport were seen due to surface nature and the uptake of zwitterion-based nanoparticles. In comparison to other insulin-containing PLA nanoparticles, zwitterion coated PLA nanoparticles induced the highest hypoglycemic effect with decreased BSL in a short time after oral administration, demonstrating the benefits of this coating on the GI uptake [11].

#### 3.2.3. Polyallylamine (PAA) Based Nanoparticles

Comb shaped amphiphilic nanoparticles, made of PAA and insulin, were developed through grafting PAA with palmitoyl pendant groups and quaternizing with methyl iodide [143]. The nanoparticles protected insulin against trypsin and pepsin, exhibiting high encapsulation efficiency. The addition of quaternary ammonium moieties to cetyl- and cholesteryl-grafted nanoparticles further increased the protection of formulation against α-chymotrypsin [144]. The opening of Caco-2 cell tight junctions was reversible and the uptake of nanoparticles in the GIT occurred by paracellular and transcellular transports, but few quantity of insulin was detected in the basal chamber because of incomplete transport across GIT and also limitations of tight junction opening [145].

#### 3.2.4. Other Polymeric Nanoparticles Containing CPP

Penetratin, a cell penetrating peptide (CPP), was modified by *bis*-β-CD to form a nanocomplex [139]. It facilitated permeation of negatively charged insulin through energy-dependent endocytosis and also energy-independent transduction through the epithelial layer. In vitro and in situ absorption studies made in both nanocomplexes have shown that modified nanocomplex induced permeation of insulin in comparison to unmodified nanocomplex. The attachment of CPP to insulin or to the nanoparticles was shown not to be essential, since penetratin grafting and also free penetratin enhance the GI absorption of insulin [146]. Self-assembled nanoparticles, made of penetratin and hydrophilic pHPMA coating, showed permeation in mucus and also epithelial uptake [140]. The hydrophilic and electrically neutral properties of pHPMA made the nanoparticles “mucus inert”, facilitating their mucus layer diffusion without being trapped. In diabetic rats, nanoparticles exhibited elevated absorption mediated by CPP in comparison to free insulin and nanoparticles administered orally reduced the BSL. Trans-activating transcriptional peptide (TAT), another CPP, was entrapped in Eudragit^®^ S100-coated chitosan nanoparticles [147]. In comparison to TAT-free nanoparticles, and mainly because of the Eudragit^®^ S100 coating, this formulation was shown to be resistance against the acidic environment of the stomach, promoting insulin absorption by TAT-mediated GI penetration, with enhanced oral bioavailability in minipigs. Low molecular weight protamine (LMWP) was currently employed as CPP and loaded in PLGA nanoparticles coated by TMC [148]. In vitro dissolution studies showed that digestive enzymes have a crucial role in the degradation rate of insulin-loaded nanoparticles. In the absorption of insulin paracellular and transcellular absorption pathways are involved because of the TMC coating and CPP [11].

#### 3.2.5. Niosomes

Niosomes are synthetic vesicles of nanometric size formed by non-ionic surfactants (e.g., alkyl-ether, esters and amides) rearranged in concentric bilayers and stabilized by cholesterol [34]. Based on their sizes and bilayers, they are classified as small unilamellar vesicles (SUV; 10–100 nm), large unilamellar vesicles (LUV; 100–3000 nm) or multilamellar vesicles (MLV) [34,149], as similar to liposomes. Niosomes can act as drug reservoirs of high drug payload to exhibit sustained or prolonged release. Their structure is made of hydrophilic, amphiphilic and also lipophilic moieties, which gives them the advantage to accommodate drugs with varying solubility. Besides that, because of their nonionic nature, niosomes are biocompatibility and of very limited toxicity [34]. Vaginal delivery of insulin-loaded niosomes was studied in vivo [150]. Span 40 and Span 60 composing niosomes loaded with insulin were produced by lipid phase evaporation technique and sonication and administered to ovariectomized alloxan induced diabetic Wistar rats. A reduction of the blood glucose with both types of niosomes was recorded, with enhanced insulin bioavailability compared to the subcutaneous administration. Niosomes exhibited prolonged insulin release followed by a hypoglycemic effect [150]. Niosomes composed of cholesterol, dicetyl phosphate and non-ionic surfactant (Span 60 or 40) were produced by thin film hydration method to load metformin [151]. Cholesterol is used in the composition of niosomes to increase the rigidity of the vesicles and to avoid the drug leakage. A prolonged release profile of these vesicles was described, following a Fickian diffusion pattern. In another study, the bioavailability of metformin-loaded niosomes was determined by measuring the serum values of glucose and drug in Wistar rats [152]. The positively charged niosomes showed a sustained release pattern in vitro and prolonged hypoglycemic effect in vivo. Repaglinide (RPG) was also incorporated in niosomes prepared from span 60 and cholesterol [153]. In vivo pharmacodynamic study in male Wistar rats demonstrated that RPG-loaded niosomes decreased blood glucose levels in comparison to the conventional dosage form. Besides, it kept RPG therapeutic level for a longer period of time. These results shown that niosomes also acted as a drug-carriers through the epithelium in deeper mucosal layers where the encapsulated drug is slowly released, having a sustained release effect. Therefore, niosomes were shown to be good delivery tool, enhancing bioavailability and reducing dosing frequency, having sustained control of hyperglycemic condition over a long time.

#### 3.2.6. Poly(amidoamine) Dendrimers

Dendrimers are nano-sized, polymeric hyperbranched macromolecules with a central core surrounded by branched monomers with different reactive end groups on the surface. Among several types, poly(amidoamine) (PAMAM) dendrimers are the most popularly used in pharmaceutical and biochemical applications [34]. PAMAM dendrimers compromised the formation of protein fibril without interfering with peptides at the tested concentration, and have shown to be appropriate delivery systems for hormones and genes in the control of hyperglycemia. PAMAM G4 has been reported to mimic the action of hypoglycemic drugs in reducing plasma hyperglycemia [154]. Major parameters (HbA1c, AOPP, AGEs and aminotransferases) have also been normalized to physiological values when animals were treated with PAMAM G4. In the diabetic animal model, PAMAM G4 dendrimers were administered by three different routes (intraperitoneally, intragastrically or subcutaneously) and evaluated their hypoglycemic effect. Labieniec-Watala et al. reported that intraperitoneal administration showed the higher blood glucose scavenging effect [155]. While intraperitoneal and subcutaneous routes were the most effective in suppressing the long-term effects of hyperglycemia, intraperitoneal injection has been associated with reduced survival rates attributed to the toxicity of the carriers. Dong et al. studied the absorption-enhancing effects of G0–G3 PAMAM dendrimers for the pulmonary delivery of insulin in rats [156]. Authors reported that PAMAMs enhanced the pulmonary absorption of insulin, being dependent both on the concentration and dendrimer generation (having G3 the greatest effect and G0 the lowest effect). The activity of lactate dehydrogenase (LDH) in bronchoalveolar lavage fluid was measured to estimate the toxicity of PAMAMs, reporting no membrane damage of lung tissues [156].

#### 3.2.7. Olymeric Micelles

Polymeric micelles are formed by self-assembled amphiphilic co-polymers in a core–shell micellar structure when their critical micellar concentration is achieved [157]. Outer functional groups allow micelle modification and the inner hydrophobic core allows hydrophobic drugs to be loaded. These systems have been used in drug delivery, because of their improved pharmacokinetics and are also able to prevent protein degradation [34]. Fang et al. produced a polyethylene glycol-phosphatidylethanolamine (PEG-PE) micelle, a negatively charged-layered hydrophilic nanocage-like structure, which managed to avoid aggregation by capturing insulin A and B chains induced by DDT and by interfering hydrophobic interaction [158]. The reduced insulin A and B chain in the nanocage recognize each other and the hypoglycemic activity analysis in mice has shown the formation of some native insulin. PEG-PE micelle was found to be an interesting artificial chaperone for in vivo and in vitro protein refolding [158]. Micelles can be stabilized by cross-linking their hydrophilic groups with a hydrophobic polymer, by producing an amphiphilic block copolymer by conjugation of multifunctional PEG with biodegradable hydrophobic polymers. The formation of phenylboronic acid-containing block copolymer and a glycopolymer complex resulted in glucose-responsive micelles. Micelle complex with PEG avoids aggregation, responds faster to the glucose changes, and is more sensitive to glucose level [159]. Micelles with stimuli responsive functional units on the surface can respond to specific stimulus signal. Through smart cargo-release behavior approach it is possible to increase efficiency of therapy and decrease side effects. Li et al. proposed self-assembled PEG-block-poly(2-dii so-propylaminoethyl methacrylate) (PEG-b-PDPA) block copolymers to develop glucose-responsive micelles [160]. Insulin, glucose oxidase and nanomicelles were co-loaded. In this formulation, insulin was released depending on glucose concentration in the microenvironment. Fast insulin release induced by pH was due to the protonation of tertiary amine groups in PDPA blocks, causing expansion of hydrophobic PDPA core and fast release of the payload. These micelles were found to be promising for the delivery of insulin [160]. Yang et al. also synthesized glucose-responsive complex micelles by self-assembly of a PBA-containing block copolymer PEG-bpoly(aspartic acid-co-aspartamidophenylboronic acid) (PEGb-P(Asp-co-AspPBA)) and a PAsp-based glycopolymer poly(aspartic acid-co-aspartglucosamine) (P(Asp-co-AGA)) [161]. The external glucose concentration was shown to be the main factor to decide the release of insulin from the complex micelles. PEG shell avoided aggregation and the self-assembly of these polymers with a hydrophilic P(Asp-co-AGA)/P(Asp-coAspPBA) allowed a faster response to control higher glucose levels, showing high glucose sensitivity by reducing the pKa of the PBA/AGA complex. PBA-based complex micelles are biodegradable and biocompatible, due to poly(aspartic acid)-based polymers together with the glycosyl moieties. For the management of diabetes, this complex micelle was shown to useful for self-regulated insulin delivery [161].

#### 3.2.8. Eudragit^®^-Based Nanoparticles

Eudragit^®^ (polymers composed of methacrylic acid esters) have also been used for the production of nanoparticles for oral delivery of insulin. It has been postulated that Eudragit^®^ nanoparticles interact with mucus layer allowing the absorption of insulin by the Peyer’s patches. These insulin-loaded nanoparticles were made with a blend of Eudragit^®^ RS (a co-polymer of methyl methacrylate, ethyl acrylate and low amount of methacrylic acid ester with quaternary ammonium groups) and poly-ε-caprolactone (PCL) [162,163]. The cationic nature of the nanoparticles obtained with this blend (Eudragit^®^ and PCL) contributed for synergistic mucoadhesive properties in the GIT, being the nanoparticles mostly absorbed by lymphatic uptake via the M cells of the Peyer’s patches in the ileum with improved glycemic response [162]. In comparison to hexameric insulin, the monomeric insulin aspart (a rapid-acting form of the peptide used in both Type 1 and Type 2 DM) showed improved absorption when both loaded in Eudragit^®^/PCL nanoparticles [163]. Insulin was protected from enzymatic degradation in the stomach due to the electrostatic interaction between insulin aspart and Eudragit^®^. Thus, by modifying the configuration of insulin it is possible to enhance biological activity. In order to form a thiomer, Eudragit^®^ L-100 can be modified with cysteine. When insulin-loaded nanoparticles were developed with thiomer and reduced glutathione, a decrease of the releasing rate of insulin in an alkali environment was observed together with the improved mucoadhesiveness of nanoparticles by forming covalent bonds with the mucus and facilitating insulin transport through the GIT [164]. Insulin-loaded polyethylene imine-based nanoparticles (coated with three layers of Eudragit^®^ NE 30D) could be absorbed in the distal part of the small intestine or colon, inducing hypoglycemic [165]. This colonic delivery system increased insulin intestinal absorption, known for its low enzymatic activity, less microvilli, less expressed P-glycoprotein and slow turnover of mucus film, and higher nanoparticle transit time. Nanoparticles coated with a pH-sensitive polymer PMV poly(methacrylic) acid co-venyl triethoxy silane have shown prevention of insulin release from the nanoparticle in acidic environment and preservation of insulin integrity [11].

### 3.3. Inorganic Nanoparticles

Inorganic nanoparticles have also been explored for oral insulin delivery [11]. In diabetic rats, insulin-loaded gold nanoparticles proved to be biocompatible and not toxic, reducing BSL by oral and intranasal administration [166]. In Caco-2 cells, gold nanoparticles capped with chondroitin sulfate showed maintained BSL with no induced cytotoxicity [167]. Chitosan can reduced gold nanoparticles, also demonstrating a hypoglycemic effect and reduced BSL [168]. Insulin can also be encapsulated in layered zirconium phosphate (ZrP) nanoparticles without pre-intercalator, however animal experiments are needed to prove their efficacy. Silica nanoparticles are known for their biocompatibility and biodegradability, larger surface area to pore volume, as well as limited size distribution for the pores [64,73,74,169]. Silica nanoparticles showed controlled release of loaded insulin with higher absorption [170]. Besides, silica nanoparticles can be modified with mucoadhesive polymers to increase their interaction with the GIT wall [64,73,74]. To reduce the loss of insulin from nanoparticles in the SGF, insulin-loaded silica nanoparticles were coated with HPMCP-55, being the BSL controlled after oral administration [170]. When coating silica nanoparticles with PEG, insulin was not retained in acid pH, showing the particles a fast release of the peptide [171]. PEGylation can indeed increase mucus diffusion, but it does not contribute to enterocytes’ absorption of nanoparticles. To trigger delivery into blood circulation insulin conjugated with low molecular weight protamine has been proposed [172].

### 3.4. Lipid-Based Nanocarriers

#### 3.4.1. Solid Lipid Nanoparticles (SLNs)

Solid lipid nanoparticles (SLNs) are submicron-sized particles (50–1000 nm) composed of a lipid crystalline core surrounded by a surfactant in aqueous dispersion [173,174]. SLNs have been reported to act as drug absorption enhancers via an oral route, as they suffer the same metabolic pathways as lipids of foods [175,176,177]. The advantages of using SLNs as drug delivery systems are associated with their physiological nature, being composed of lipids similarly resembling those of the human body offering high biocompatibility and biodegradability [178,179,180]. Different SLN formulations composed of cetyl palmitate, glyceryl monostearate or glyceryl palmitostearate as solid lipid, and using poly-oxyethylene esters of 12-hydroxystearic acid and lecithin as surfactants, have been produced for the loading of insulin [181]. Although all the three SLN formulations exhibited a burst release within the first 4 h, glyceryl palmitostearate-SLN showed the lowest burst release in vitro (33.28%) and the highest pharmacological activity in vivo. Insulin-loaded SLNs were developed based on Softisan^®^100 (hydrogenated coco-glycerides) [68]. Toxicity was evaluated using *Drosophila melanogaster* model, showing that neither SLNs nor the bulk lipid were toxic. Surface of SLNs, composed of stearic acid and stabilized by polyxamer and lecithin combination, was modified using lectin and also wheat germ agglutinin [182]. Despite the protection of insulin against enzymatic degradation, the oral bioavailability could only be improved by the use of protease inhibitors. It is known that hydrophobic nature of solid lipid matrix does not allow a high encapsulation efficiency of hydrophilic peptides, such as insulin. To enhance the encapsulation efficiency of insulin various approaches have been proposed based on hybrid lipid-polymeric systems: (i) using hydrophilic polymers (hydrophilic METHOCEL™ Cellulose Ethers and PLGA), which improved emulsion stability during w/o/w double emulsion preparation, leading to an improvement of insulin encapsulation efficiency [183]; (ii) adding an agent to control the viscosity (e.g., PEG), which may preserve the peptide bioactivity in the GIT and improve the encapsulation efficiency during the production of nanoparticles [184] and (iii) producing hybrid nanoparticles using the novel reverse micelle-double emulsion method [185]. The reverse micelle-double emulsion method has shown high efficiency to encapsulate insulin, reducing the risk of insulin leakage from the nanoparticles over storage time. Another study reported the loading of insulin in the SLNs obtained by a complex of insulin and phospholipids with high encapsulation efficiency [186]. Zhang et al. reported the use of SLNs composed of stearic acid and octa-arginine to enhance insulin oral delivery, with a significant hypoglycemic effect in diabetic rats [187].

#### 3.4.2. Nanostructured Lipid Carriers (NLCs)

Nanostructured lipid carriers (NLCs) differ from SLN as the former are based on a blend of solid and liquid lipids, which together remain solid at body and room temperature. NLCs offer advantages over SLNs as higher loading capacity for lipophilic compounds and improved drug release modeling [188]. Essential oils with hypoglycemic properties have been recently proposed as active ingredients of NLCs of the management of DM [189]. Other poorly-water phyto ingredients with anti-hyperglycemic effects have been successfully loaded in NLCs to improve their oral bioavailability. Baicalin, a flavone glycoside, has been loaded in NLCs produced from Precirol and Miglyol aiming to improve its antidiabetic effects in the rat model. The particles showed sustained release and enhanced antihyperglycemic effects of baicalin by inhibiting lipid peroxidation, when loaded into NLC [190,191]. Quercetin, thymoquinone and resveratrol have also been loaded into NLCs which offered sustained-release of the loaded drugs and improved antidiabetic effects [42]. Using phase inversion method, quercetin was encapsulated in NLCs showing sustained release and higher bioavailability [192]. Cationic NLCs were loaded with quercetin with high bioavailability in lung, kidney and liver [193].

#### 3.4.3. Liposomes

Liposomes are small vesicles composed of one or more phospholipid bilayers produced from natural non-toxic phospholipids and cholesterol [194]. They are used in drug delivery, because they are biodegradable, biocompatible, have low toxicity, capacity to entrap lipophilic and hydrophilic drugs and are useful for site-specific/targeted delivery [34]. Many approaches have been proposed using liposomes to decrease drug toxicity of loaded drugs and to target specific cells to enhance efficacy and safety [195]. Zhang et al. developed inulin-loaded liposomes modified with targeted ligand biotin for oral delivery. Liposomes were produced by incorporation of biotin-1,2-distearoyl-*sn*-glycero-3-phosphatidylethanolamine (DSPE) in the lipid bilayer of liposome [196]. Lipid:cholesterol ratio of 3:1 reduced the risk of insulin leakage from internal aqueous compartments. The hypoglycemic effect of insulin-loaded liposomes was shown to be dependent on the biotin-DSPE ratio and mean size of the carriers. Indeed, the smaller size of liposomes promoted a higher uptake by receptor-mediated endocytosis through intestinal epithelia. In comparison to conventional liposomes, biotin-DSPE liposomes improved insulin bioavailability [196].

The coating of liposomes with chitosan has also been proposed to reduce the insulin uptake in the gut attributed to the enhanced mucoadhesiveness of the particles and higher zeta potential promoted by the positively charged polysaccharide [197]. These liposomes were orally administered to Kunming mice, demonstrating to be as effective as parenteral insulin in enhancing the hypoglycemic effect. In order to enhance formulation’s gastric residence time, these liposomes were protected from pepsin and trypsin. At neutral pH, chitosan molecule shows a coiled configuration, fixing on liposomes surface and thus creating a protective layer [197]. Agrawal et al. developed folic acid functionalized liposomes coated with anion polyacrylic acid and cation polyallyl amine hydrochloride for oral administration of insulin [198]. In comparison to conventional liposomes, studies on Caco-2 cell and ex vivo gastroenteric uptake shown enhanced uptake of functionalized liposomes. In comparison to subcutaneous insulin administration, pharmacodynamic studies of orally administered folic acid liposomes shown enhanced hypoglycemic effect and higher insulin bioavailability, in comparison to the standard subcutaneous form. Subcutaneous insulin is associated with drastic decrease of blood glucose levels, whereas the oral administration of recombinant human insulin loaded with functionalized liposomes provided a more sustained hypoglycemia with less side effects. Hu et al. compared the stability in simulated GI media and ex vivo GI media from rats of oral liposomes composed of glycocholate in comparison with conventional liposomes based on soybean phosphatidylcholine and cholesterol [199]. Prevention of insulin leakage from liposomes and its proteolytic degradation was enhanced with the presence of the bile salt in the vesicles. Using a fluorescent dye (calcein), Niu et al. showed that at low pH environment calcein was rapid release of from sodium glycocholate composed liposomes, protecting loaded insulin from enzymatic degradation. Liposomes composed of bile salts promote gut insulin absorption with improved oral bioavailability of the peptide, in comparison to conventional liposomes [200]. Liposomes can be thus a good choice for the delivery of peptides (insulin, GLP-1) and other sugar lowering drugs, allowing for a better control of the hyperglycemic stage through alternative administration routes [3,34].

The leaves of *Orthosiphon stamineus* are commonly used in Southeast Asia and Europe as herbal tea for their diverse beneficial effects in human health, with known diuretic, hypouricemic, hepatoprotective, antiangiogenic, anticancer, antioxidant, antimicrobial and also hypoglycemic activities [201]. The solubility of its ethanolic extract was significantly enhanced when loaded in liposomes composed of soybean phospholipids with improved free-radical-scavenging activity in comparison to the free extract.

#### 3.4.4. Nanoemulsions

Nanoemulsions are also known to enhance the delivery of lipophilic compounds with antidiabetic properties [42]. Bitter gourd seed oil nanoemulsions containing α-eleostearic acid were orally administered to a diabetic rat model with improved antidiabetic properties [202], higher cellular uptake and antioxidant effects [203]. Streptozotocin-induced diabetic rat model were treated with alpha-tocopherol-loaded nanoemulsions, which showed protective effect in various organs [204]. Li et al. produced insulin-loaded nanoemulsions coated with chitosan/alginate by polyelectrolyte cross-linking [205]. Conformational stability of insulin during cross-linking was confirmed while nanoemulsions kept their integrity during the in vitro leakage study in simulated gastric medium. Hypoglycemic effects were observed in both normal and diabetic rats. A significantly prolonged hypoglycemic effect after oral administration of the coated nanoemulsions compared with subcutaneous insulin was also demonstrated.

### 3.5. Drug Nanosuspensions

Drug nanosuspensions (drug nanocrystals) are another type of delivery system that can be exploited for oral administration, in which active ingredients are in the solid state, have a particle size up to 1 µm surrounded by a hydrophilic surfactant in aqueous dispersion [206]. Drug nanosuspensions have been proposed to improve the solubility of Biopharmaceutical Classification System (BCS) II and IV drugs. Vaculikova et al. improved the solubility of the poorly water soluble glibenclamide—an oral hypoglycemic BCS class II drug that stimulates pancreatic beta cells to secrete insulin—by producing nanosuspensions by emulsion solvent evaporation method using dichloromethane as solvent and carboxymethyl dextran sodium salt as stabilizer [207]. Glimepiride is another BCS class II drug, which was formulated as nanosuspensions with improved solubility [208]. Nicotinamide-streptozotocin-induced diabetic rat models were treated with the glimepiride nanosuspensions with improved for pharmacokinetic and antihyperglycaemic activity. Phyto-ingredients have been formulated in nanosuspensions to improve the antidiabetic efficacy in comparison to classical formulations [42]. Gymnemic acids were prepared using a nanosuspension method, also showing improved bioavailability [209]. The authors also studied the effect of these nanosuspensions in humans, showing an enhanced antidiabetic effect and higher glucose-lowering effect [210]. Berberine nanosuspensions (antidiabetic compound) have shown an enhanced antidiabetic effect in T2DM animal models with a lower dosage level [211].

## 4. Conclusions

Subcutaneous injection of insulin remains the conventional pharmacotherapeutic approach regardless of the diagnosed type of DM. Injections are associated however to the patient’s non-compliance due to discomfort, pain and risk of local infection. The exploitation of other friendlier, non-invasive administration routes (e.g., oral, nasal and ocular) led to the development of a diversity of nanoparticles aiming at protecting insulin, increasing its physicochemical stability in vivo, enhancing its absorption and thereby its bioavailability. Oral delivery of insulin would be preferential as it mimics pharmacokinetics of endogenous insulin. Not only insulin has already been successfully loaded into nanoparticles but also other sugar lowering drugs for the treatment of diabetes and its complications. Table A1 summarizes several types of nanoparticles used for the loading of insulin and other sugar lowering drugs, and their reported effects in vivo. Nanoparticles may be tailored to open tight junctions (promoting paracellular absorption) as well as to site-specific targeting receptors (promoting transcellular pathway). To further improve insulin uptake in the GIT, different targeting ligands (vitamin B12, folate, transferrin and CPP) have also been exploited for surface tailoring nanoparticles. New strategies based on nanoparticles are now in the pipelines for the chronic treatment of diabetes—the epidemic disease of the 21st century.

## Appendix A

**Table A1:** Nanoparticulate delivery systems described for the treatment of Diabetes Mellitus.

Type of Delivery System	Loaded Bioactive/Drug	Administration Route	In vivo Effects
Insulin-loaded chitosan nanoparticles	Insulin	Oral	BSL reduction Prolonged effect Biodistribution (SPECT): stomach, small and large intestine, kidney, urinary bladder
Insulin-loaded alginate nanoparticles	Insulin	Oral	BSL reduction Prolonged hypoglycemic effect
Insulin-loaded dextran nanoparticles	Insulin	Oral	BSL reduction Prolonged hypoglycemic effect
Insulin-loaded PLGA nanoparticles	Insulin	Oral	BSL reduction Prolonged hypoglycemic effect
Insulin-loaded PLA nanoparticles	Insulin	Oral	BSL reduction Prolonged hypoglycemic effect biodistribution: spleen, kidney, liver, heart, lungs
Insulin-loaded PAA nanoparticles	Insulin	Oral	Only in vitro studies in Caco-2 cell line
Insulin-loaded nanoparticles containing CPP	Insulin	Oral	BSL reduction
Insulin-loaded inorganic nanoparticles and Insulin-loaded nanoparticles containing Eudragit^®^	Insulin	OralNasal	BSL reduction Maximal hypoglycemic effect No toxicity in Zebrafish
Insulin-loaded SLN	Insulin	Oral	BSL reduction
Liposomes	Insulin Metformin Calcein GLP-1	Oral	Hypoglycemic effect Enhance absorption of insulin Maximum oral bioavailability
Niosomes	Insulin Metformin Metformin hydrochloride Repaglinide Pioglitazone Gliclazide	Oral	Enhance insulin permeation Enhance bioavailability
Dendrimers	Human and bovine pancreatic insulin Calcitonin	Subcutaneous	Enhance glucoregulatory effects
Micelles	Lyophilized human and porcine insulin Insulin	Oral	Prevention of aggregation of insulin Enhance bioavailability
